# On the Minute Attention to the Comforts of the Insane

**Published:** 1848-06

**Authors:** 


*On the Minute Attention to the Comforts of the Insane.*—If it be necessary for the treatment of pauper patients that such especial attention should be paid to these points, *à fortiori*, how absolutely indispensable is it that patients of a higher class, confined in private asylums, should have around them all the comforts and little elegancies of life to which they have been accustomed when well, and whilst at home! It is impossible to be too careful in directing that all the service of the table should be in accordance with the habits of the patients. The sense of banishment from home, and of confinement, and the consciousness of mental infirmity and dependence, are mitigated in the mind of many a silent, uncomplaining patient, by these means. Among the depressing recollections of the insane of the higher classes, when recovering from insanity, none are more frequent, or felt to be more degrading, than those connected with any want of respect shown to them, or any disregard of decent customs as to their meals. Yet, without great attention, they will sometimes be found, when quite well enough to appreciate what is done, sitting down to a dinner of meat, vegetables, and pudding, all sent to them on one plate. Negligences of this kind produce fretfulness and discontent, and tend to retard convalescence. If attendants are allowed to practise this kind of negligence, they soon fall into habits of rudeness, and even of inhumanity, fancying that the patients do not observe their conduct, and that their feelings are of no consequence.—*Journal of Psychological Medicine.*

